# Berberine Prolongs Mouse Heart Allograft Survival by Activating T Cell Apoptosis *via* the Mitochondrial Pathway

**DOI:** 10.3389/fimmu.2021.616074

**Published:** 2021-02-25

**Authors:** Yunhan Ma, Guoliang Yan, Junjun Guo, Fujun Li, Haiping Zheng, Chenxi Wang, Yingyu Chen, Yuhan Ye, Helong Dai, Zhongquan Qi, Guohong Zhuang

**Affiliations:** ^1^Fujian Provincial Key Laboratory of Organ and Tissue Regeneration, School of Medicine, Organ Transplantation Institute, Xiamen University, Xiamen, China; ^2^School of Medicine, Xiamen University, Xiamen, China; ^3^Department of Anesthesiology, First Affiliated Hospital of Harbin Medical University, Harbin, China; ^4^Department of Pathology, Zhongshan Hospital Affiliated to Xiamen University, Xiamen, China; ^5^Department of Kidney Transplantation, Center of Organ Transplantation, The Second Xiangya Hospital of Central South University, Changsha, China; ^6^Clinical Research Center for Organ Transplantation in Hunan Province, Changsha, China; ^7^Clinical Immunology Center, Central South University, Changsha, China; ^8^Medical College, Guangxi University, Nanning, China

**Keywords:** alloimmunity and transplantation, transplantation immunology, immunoregulation, immunological tolerance and memory, T cell, berberine, heart allograft survival

## Abstract

Berberine, which is a traditional Chinese medicine can inhibit tumorigenesis by inducing tumor cell apoptosis. However, the immunoregulatory of effects berberine on T cells remains poorly understood. Here, we first examined whether berberine can prolong allograft survival by regulating the recruitment and function of T cells. Using a major histocompatibility complex complete mismatch mouse heterotopic cardiac transplantation model, we found that the administration of moderate doses (5 mg/kg) of berberine significantly prolonged heart allograft survival to 19 days and elicited no obvious berberine-related toxicity. Compared to that with normal saline treatment, berberine treatment decreased alloreactive T cells in recipient splenocytes and lymph node cells. It also inhibited the activation, proliferation, and function of alloreactive T cells. Most importantly, berberine treatment protected myocardial cells by decreasing CD4^+^ and CD8^+^ T cell infiltration and by inhibiting T cell function in allografts. *In vivo* and *in vitro* assays revealed that berberine treatment eliminated alloreactive T lymphocytes *via* the mitochondrial apoptosis pathway, which was validated by transcriptome sequencing. Taken together, we demonstrated that berberine prolongs allograft survival by inducing apoptosis of alloreactive T cells. Thus, our study provides more evidence supporting the potential use of berberine in translational medicine.

## Introduction

Organ transplant patients are prescribed several immunosuppressive drugs to avoid immune recognition and rejection of the allogeneic organs. However, global immunosuppressive drug administration might also cause severe side effects, such as tumorigenesis, acute renal toxicity, diabetes, and hyperlipidemia (1–5). Therefore, it is necessary to develop new immunosuppressive drugs with fewer side effects and the capacity to inhibit transplant rejection. Screening the existing antineoplastic drugs for immunosuppressive potential is a feasible strategy to quickly identify such drugs.

Berberine, an isoquinoline alkaloid extracted from Rhizoma coptidis and Cortex Phellodendri, exerts multiple biological and pharmacological effects and it has long been utilized for the treatment of tumorigenesis owing to its capacity to induce tumor cell apoptosis (6–8). Berberine might exert a similar effect on alloreactive T cells and benefit organ transplant recipients. In animal liver transplantation, the addition of berberine to the preservation solution can protect the mitochondrial function of the graft from ischemia–reperfusion injury (9), which likely benefits allograft survival. Moreover, berberine has an anti-inflammatory and immunomodulatory effect with hyperlipidemia (10), diabetes (11), rheumatoid arthritis (12, 13), and cardiac dysfunction (14). Recent studies have also demonstrated that it induces dendritic cell apoptosis (12) and suppresses the production of the proinflammatory cytokines tumor necrosis factor-alpha (TNF-α) (14), interleukin (IL)-1 (15), and IL-6 (16). Due to the wide availability and low cost of berberine in China, it might represent a potential solution to the side effects and affordability issues associated with organ transplantation.

Despite the emerging evidence of the potential role of berberine in treating various diseases, neither its influence on T cells nor its immunosuppressive potential *in vivo* has been investigated. Therefore, here, we investigated the efficacy of berberine in a mouse cardiac transplantation model. We demonstrated that treatment with a moderate (5 mg/kg), but not high, dose of berberine significantly prolonged mouse vascularized cardiac allograft survival with no toxic side effects. *In vivo* and *in vitro* data suggested that berberine inhibits the activation and proliferation of CD4^+^ and CD8^+^ T cells and induces T cell apoptosis by activating the mitochondrial pathway. These findings suggest that berberine is a viable immunosuppressive agent for experimental organ transplantation.

## Materials and Methods

### Animals and Drugs

Male C57BL/6 (H-2^b^) and BALB/c (H-2^d^) mice aged 10–12 weeks were purchased from Shanghai SLAC Laboratory Animal Co., Ltd (Shanghai, China) and housed under specific pathogen-free conditions in accordance with the guidelines of the Animal Care and Use Committee and Ethics Committee of Xiamen University (Committee’s reference number: XMULAC20170243). Berberine (purity > 98%) was purchased from Sigma-Aldrich (MO, USA) (17).

### Heterotopic Cardiac Transplantation

Mouse heterotopic heart transplantation was performed as previously described (18–20). BALB/c mouse hearts were transplanted into the necks of C57BL/6 mice. Rejection was defined as the complete cessation of heart contractions and was confirmed by neck palpation.

### Flow Cytometry

To measure T cell apoptosis, 1 × 10^6^ splenocytes (SPCs) and lymph node cells (LNCs) were prepared and stained with CD4^+^-APC and CD8^+^-PE/CY7 (eBioscience, San Diego, CA, USA) for 30 min at 4°C. After washing with PBS, the cells were stained with Annexin V and propidium iodide according to the manufacturer’s instructions (Dojindo Laboratories, Japan) and analyzed by flow cytometry. To measure T cell activation, the SPCs and LNCs were freshly stained with CD4^+^-APC, CD8^+^-PE/CY7, CD44^+^-PE, and CD69^+^-PE (eBioscience) for 30 min at 4°C and analyzed by flow cytometry. Isotype control staining was also performed as described (21). Data were analyzed using FlowJo software version 10 (Tree Star Inc., Ashland, OR, USA).

### T Cell Proliferation Assays and Measurement of Cytokines

T cells were labeled with 5 μmol/L CFSE proliferation dye (Invitrogen, Carlsbad, CA, USA) and cultured in the presence of an anti-CD28 antibody (0.5 μg/ml)- and anti-CD3 antibody (1 μg/ml)-coated plate as per the manufacturer’s instructions (eBioscience). After 72 h, cells were harvested, stained with CD4^+^-APC and CD8^+^-PE/CY7 for 30 min at 4°C and analyzed by flow cytometry. Cell divisions were demarcated according to CFSE staining brightness. The levels of IFN-γ in the supernatant were measured using ELISA according to the manufacturer’s instructions (Boster, Wuhan, China), and the absorbance was read at 450 nm using a microplate spectrophotometer (Thermo Fisher Scientific, Waltham, MA, USA).

### Mixed Lymphocyte Reaction

SPCs from naïve BALB/c mice treated with mitomycin C (40 μg/ml) were used as stimulator cells, whereas the SPCs from the recipient C57BL/6 mice were used as the responder cells. Totals of 1 × 10^6^ stimulator and 2 × 10^5^ responder cells were added to a 96-well round-bottom plate and cultured at 37°C for 72 h. Cell proliferation was measured using the BrdU kit (Roche, Mannheim, Germany), and each experiment was performed in triplicate.

### Transcriptome Sequencing (RNA-seq)

SPCs were collected at post-operative day (POD) 7, and the total RNA was isolated using TRIzol (Transgen Biotech, Beijing, China) reagents. RNAseq was carried out *via* a commercially available service (service ID# F19FTSECWLJ6127, BGI, Huada Gene, China). The raw sequencing data reported in this manuscript have been deposited in the NCBI Sequence Read Archive (SRA) and are accessible through SRA accession number: PRJNA689623, or https://www.ncbi.nlm.nih.gov/sra/PRJNA689623. The sequencing library was used for cluster generation and sequencing on the BGISEQ-500 system. For gene expression analysis, the matched reads were calculated and then normalized based on FPKM. Fold-changes were calculated for all possible comparisons and a 2-fold cutoff was used to identify the gene expression changes (22).

### Histology and Immunofluorescence Staining

The allograft was fixed in 10% phosphate-buffered formalin, embedded in paraffin, and cut into 5-μm sections for hematoxylin‐eosin (HE) staining. The sections were then incubated with primary antibodies including anti‐CD4 Ab (Boster), anti‐CD8 Ab (Boster), anti-CD3 (Abcam Inc, Cambridge, MA, USA), anti-ki67 (Affinity, Changzhou, China), anti-actinin (Boster), anti-cleaved-caspase-3 (Affinity), anti-cleaved-PARP (Affinity), anti-IFN-γ (Affinity), and goat anti-rabbit IgG secondary antibodies (Boster). Images were captured with a Leica Aperio Versa 200 (Germany) whole slide imaging system. Two cardiologists blinded to the experimental conditions graded acute rejection according to the International Society of Heart and Lung Transplantation criteria (23).

### Quantitative Real-Time PCR

The mRNA levels of inflammatory and cytotoxic genes were measured by qPCR. mRNA was isolated and reverse transcribed into cDNA using the High Capacity cDNA Reverse Transcription Kit (Applied Biosystems, Thermo Fischer Scientific). The following primers were used: IFN-γ forward 5-CGGCACAGTCATTGAAAGCCTA-3; IFN-γ reverse, 5ʹ-GTTGCTGATGGCCTGATTGTC-3ʹ; FasL forward, 5ʹ-GCCCATGAATTACCCATGTCC-3ʹ; FasL reverse, 5ʹ-ACAGATTTGTGTTGTGGTCCTT-3ʹ; Bcl-2 forward, 5ʹ-TCCTTCCAGCCTGAGAGCAACC-3ʹ; Bcl-2 reverse, 5ʹ-TCACGACGGTAGCGACGAGAG-3ʹ; TNF-α forward, 5ʹ-CATCTTCTCAAAATTCGAGTGACAA-3ʹ; TNF-α reverse, 5ʹ-TGGGAGTAGACAAGGTACAACCC-3ʹ; β-actin forward, 5ʹ-CATCCGTAAAGACCTCTATGCCAAC-3ʹ; β-actin reverse, 5ʹ-ATGGAGCCACCGATCCACA-3ʹ. Relative gene expression was calculated *via* the −2^ΔΔCt^ method.

### Western Blotting

Grafts and SPCs were lysed in fresh extraction buffer (Sigma-Aldrich, St. Louis, MO, USA) supplemented with a protease inhibitor and phosphatase inhibitor (Gold Biotechnology, St. Louis, MO, USA). The extracted proteins were separated on a 15% SDS-polyacrylamide gel and electroblotted onto a polyvinylidene fluoride membrane. The membrane was blocked using 5% skim milk-containing TBST for 60 min at 20–25°C and incubated with primary antibodies against Bcl-2 (Affinity), Bax (Affinity), cytochrome c (Affinity), cleaved-caspase-3(Affinity), cleaved-PARP (Affinity), and PCNA (Abcam) overnight at 4℃. The membranes were then washed with TBST and incubated with goat anti-mouse IgG (R&D Systems, Inc., Minneapolis, MN, USA) and goat anti-rabbit IgG (R&D Systems) secondary antibodies. Bound antibodies were detected using an electrochemiluminescence detection system (Amersham Life Science, Arlington Heights, IL, USA). β-actin was used as the loading control.

### Statistical Analysis

All data were analyzed using GraphPad Prism version 6.00 (GraphPad Software, San Diego, CA, USA) using the Student’s *t*-test or one-way analysis of variance (one-way ANOVA) followed by Tukey’s test for multiple comparisons. The results were considered statistically significant for p < 0.05, and the values are presented as the mean ± SEM.

## Results

### Berberine Is Safe for Heart Transplant Experiments

**Figure 1:**
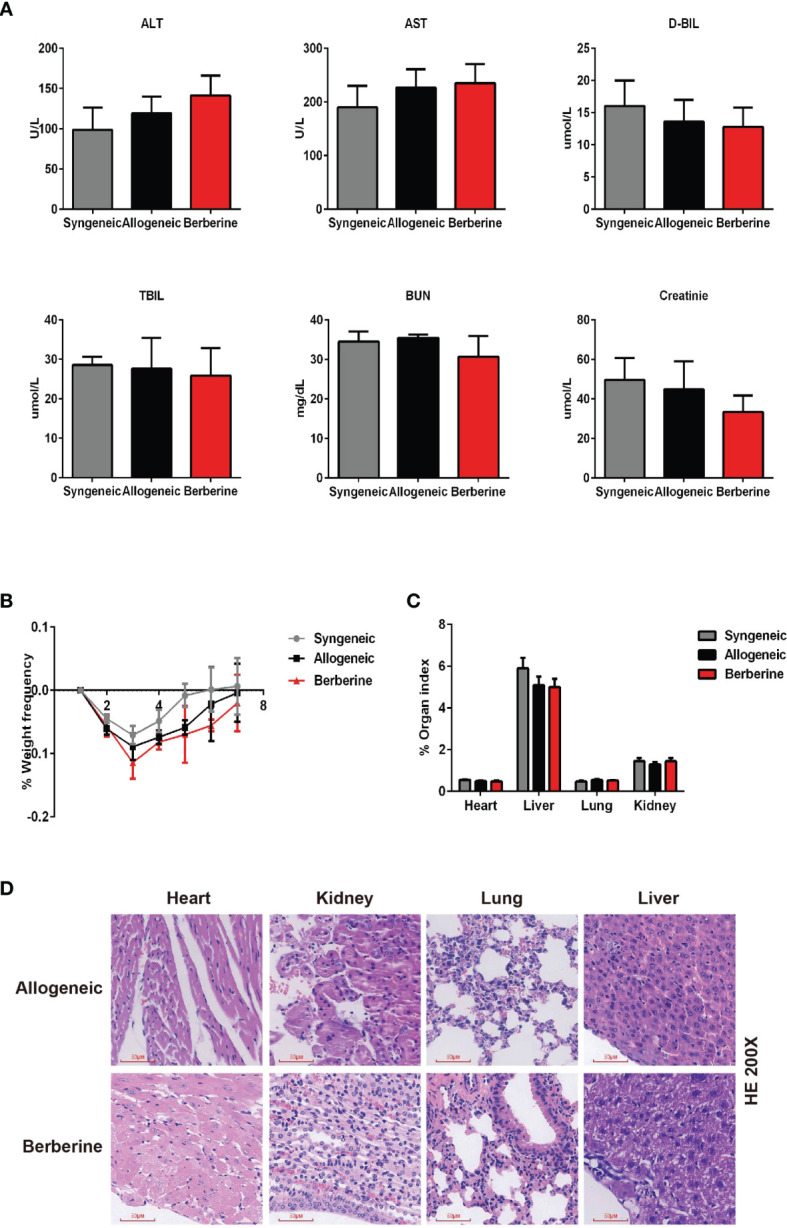
Berberine safety for heart transplant experiments. BALB/c heart grafts were transplanted into C57BL/6 mice at POD 0. **(A)** Renal function and hepatic function of transplant recipient mice measured from serum. **(B)** Body weight changes in transplant recipient mice. The weight changes in berberine (5 mg/kg)-treated and normal saline-treated recipients was assessed daily from POD 0 to POD 7. Syngeneic transplant recipients are shown for comparison (n = 5 mice/group). **(C)** Organ indexes in transplant recipient mice. The heart, liver, lungs, and kidneys of recipient mice were harvested and weighed at POD 7 (n = 5 mice/group). **(D)** HE staining of heart, liver, lung, and kidney collected at POD 10 from normal saline (Allogeneic) and berberine (5 mg/kg) -treated recipient mice (scale bar = 50 μm; original magnification: ×200). POD, post-operative day; HE, hematoxylin and eosin; AST, aspartic aminotransferase. ALT, alanine aminotransferase. TSB, total serum bilirubin. DSB, direct serum bilirubin.

To determine the toxicity of berberine for mouse cardiac transplantation, BALB/c mouse hearts were transplanted into C57BL/6 mice, which were treated with various concentrations of berberine intraperitoneally. Low (2.5 mg/kg) and moderate (5 mg/kg) doses of berberine did not significantly influence recipient survival. However, the recipient mortality rate increased with an increase in the berberine concentration to > 5 mg/kg. All recipients suffered perioperative deaths after administration of berberine at a dose of 10 mg/kg (**Table 1**). Based on these findings, transplant recipients were treated with a moderate dose (5 mg/kg) of berberine for 10 days; thereafter, blood samples were collected and physiological and biochemical indicators were monitored. Compared to those in syngeneic recipients, renal and hepatic function-related aspartic aminotransferase, alanine aminotransferase, total serum bilirubin, direct serum bilirubin, urea, and creatinine parameters were not significantly different among treatments, indicating that a moderate dose of berberine exhibits no renal and hepatic toxicity (**Figure 1A**). The body (**Figure 1B**) and organ (**Figure 1C**) weights were also equivalent among the treatment groups. Furthermore, following HE staining at POD 10, we found that the recipients’ major organs had normal structures (**Figure 1D**), indicating that appropriate doses of berberine exhibit low toxicity and could be used safely for mouse heart transplantation.

**Table 1:** Transplant recipient mice survival rate following berberine treatment^a^.

Transplant recipient mice survival rate following berberine treatment				
Berberine dose (mg/kg/d)	Survival time of recipient (days)			
2.5	7	10	10	10
5	15	17	21	39
7.5	4	4	5	35
10	3	3	4	4

^a^C57BL/6 recipients were treated with 0, 2.5, 5, 7.5 or 10 mg/kg (i.p.) of berberine at POD 0, 2, 4, 6, 8, and 10 (n = 4 mice/group). Survival of berberine-treated recipients was confirmed by direct visual examination (Red font indicates the death of recipient).

### Berberine Prevents Acute Rejection of Fully Mismatched Cardiac Allografts

**Figure 2:**
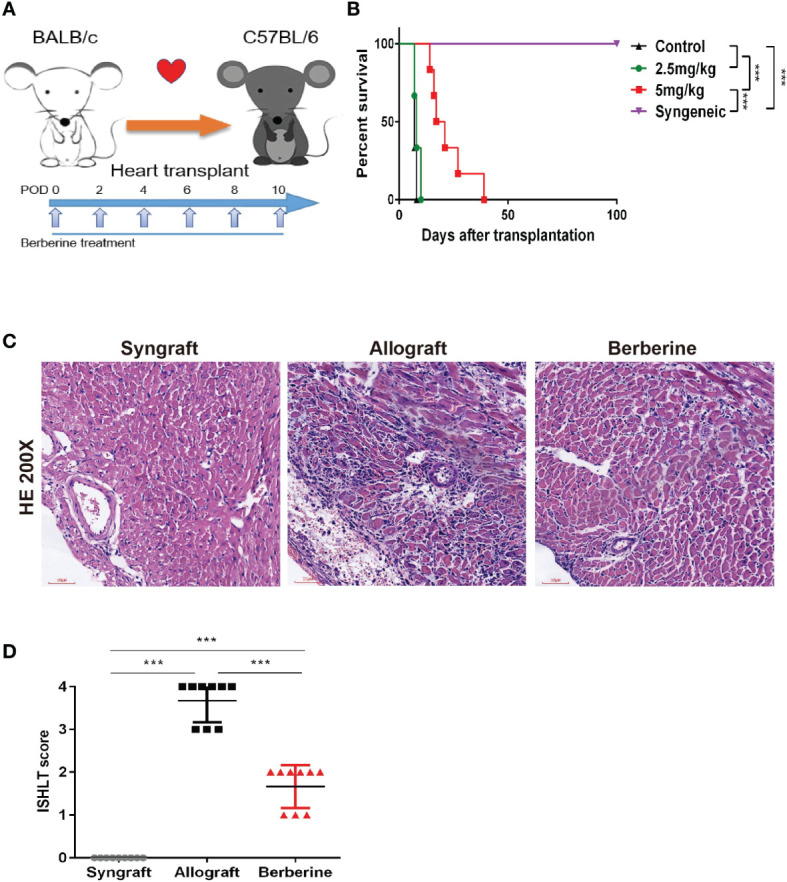
The effect of berberine on acute rejection of fully mismatched cardiac allografts. **(A)** Treatment schedule for berberine. BALB/c cardiac grafts were transplanted into C57BL/6 recipients at POD 0. C57BL/6 recipients were treated with normal saline, 2.5 mg/kg berberine, or 5 mg/kg of berberine i.p. on the days indicated by arrows. **(B)** Survival of cardiac allografts in berberine-treated and normal saline-treated mice recipients. A syngeneic transplant is shown for comparison (n = 6 mice/group). **(C)** HE staining of cardiac allografts collected at POD 7 from berberine-treated and normal saline-treated mouse recipients. A syngeneic graft at POD 100 is shown for comparison (scale bar = 50 μm; original magnification: ×200). **(D)** ISHLT grades from nine different sections from three heart allografts in each group. HE, hematoxylin and eosin; POD, post-operative day. ***p < 0.001 compared to the normal saline-treated group.

To determine the effect of berberine in a BALB/c-to-C57BL/6 mouse heart transplantation model, recipients were treated with low (2.5 mg/kg) and moderate doses (5 mg/kg) of berberine or normal saline from POD 0 to POD 10 using a dosing regimen, as shown in **Figure 2A**. We found that the administration of moderate doses of berberine significantly prolonged heart allograft survival compared to that with both low doses and normal saline treatment [median survival time = 19 (moderate dose) days vs. 8 days (low dose) and 7 days (normal saline), *p* < 0.05] (**Figure 2B**). The recipients of syngeneic heart transplants survived for more than 100 days. Next, we investigated the protective effect of moderate doses of berberine on allografts. Following berberine treatment, the allografts presented with near normal myocardial fiber histology at POD 7, whereas allografts from normal saline-treated mice showed severe cellular infiltration and myocardial destruction (**Figure 2C**) and were characterized by a higher International society for Heart and Lung Transplantation rejection score (**Figure 2D**). Syngeneic grafts at POD 100 showed normal myocardial structure without inflammatory cell infiltration.

### Berberine Treatment Decreases T Cell Immune Responses to Cardiac Allografts

**Figure 3:**
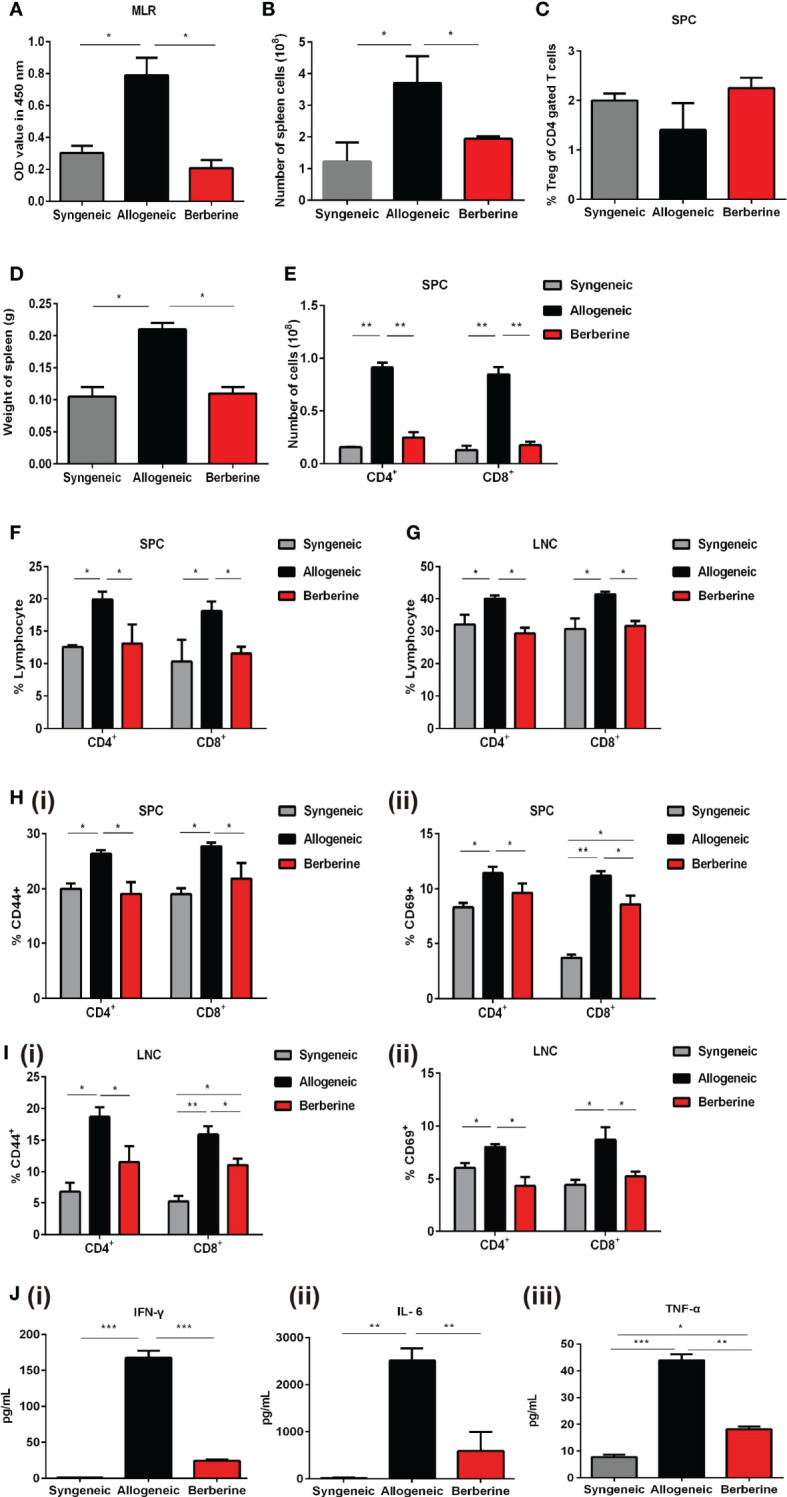
Effect of berberine treatment on the T cell immune responses to cardiac allografts. **(A)** MLR responses. Recipient SPCs were isolated at POD 7 (responders) and mitomycin C-treated naïve BALB/c SPCs (stimulators) were co-cultured for 3 days (n = 3 mice/group). Total SPCs were isolated at PDO 7, and the absolute numbers of **(B)** SPCs were determined by flow cytometry. **(C)** The percentage of CD4^+^Foxp3^+^Treg cells was determined by flow cytometry. Syngeneic recipients are shown for comparison (n = 3 mice/group). **(D)** The spleens of recipient mice were harvested and weighed at POD 7 (n = 5 mice/group). **(E)** The absolute numbers of CD4^+^ and CD8^+^ T cells SPCs (n = 3 mice/group). **(F)** SPCs and **(G)** LNCs were isolated at POD 7, and the percentage of CD4^+^ and CD8^+^ T cells was determined by flow cytometry. Syngeneic recipients are shown for comparison (n = 3 mice/group). **(H)** CD44^+^CD69^+^ T cell activation assays. SPCs were isolated at POD 7, and the percentages of (i) CD4^+^CD44^+^CD69^+^ and (ii) CD8^+^CD44^+^CD69^+^ T cells were determined by flow cytometry (n = 3 mice/group). **(I)** LNCs were isolated at POD 7, and the percentages of (i) CD4^+^CD44^+^CD69^+^ and (ii) CD8^+^CD44^+^CD69^+^T cells were determined by flow cytometry (n = 3 mice/group). **(J)** Serum plasma levels of proinflammatory cytokines. Peripheral blood was collected at POD 7, and (i) IFN-γ, (ii) IL-6, and (iii) TNF-α plasma levels were measured by ELISA (n = 3 mice/group). SPCs, spleen cells; LNCs, lymph node cells; MLR, mixed lymphocyte reaction; POD, post-operative day. *p < 0.05, **p < 0.01, ***p < 0.001 compared to the normal saline-treated group.

To evaluate the immunosuppressive activity of berberine in mice after heart transplantation, recipient SPCs were harvested and incubated with mitomycin C-pre-treated BALB/c SPCs. This assay demonstrated that berberine treatment significantly reduced SPC proliferative responses to allo-antigens compared to that with normal saline treatment, and no significant difference was observed between berberine-treated and syngeneic transplant recipients (**Figure 3A**). Compared to those with normal saline treatment, the absolute numbers of SPCs (**Figure 3B**) and the weights of spleens (**Figure 3D**) were significantly lower in berberine-treated mice but similar to those in syngeneic transplant recipients. Similar results were obtained when investigating the number of CD4^+^ and CD8^+^ T cells (**Figures 3E–G**). However, the proportion of Tregs within the CD4 compartment among SPCs was not significantly different between each treatment (**Figure 3C**). To monitor T cell activation, recipient SPCs and LNCs were incubated with CD44^+^ and CD69^+^ cells. Compared to that with normal saline treatment, CD4^+^/CD8^+^ CD44^+^CD69^+^ T cell activation was significantly inhibited in recipient SPCs (**Figure 3H**) and LNCs (**Figure 3I**) following berberine treatment (p < 0.05). We further evaluated whether berberine would regulate the production of proinflammatory cytokines. Transplant recipient serum was collected at POD 7, and IL-6, IFN-γ, and TNF-α protein secretion was measured by ELISA. Decreased levels of serum proinflammatory cytokines IFN-γ, IL-6, and TNF-α were observed in the berberine-treated recipients compared to levels in recipients treated with normal saline. Furthermore, similar serum proinflammatory cytokine levels were observed between syngeneic and berberine-treated recipient mice (**Figure 3J**). These findings suggest that berberine treatment regulates cell-mediated acute rejection by decreasing the number of effector T cells and suppressing their function.

### Myocardial Cell-Protective Effects of Berberine Treatment Are Associated With Reduced T Cell Infiltration

**Figure 4:**
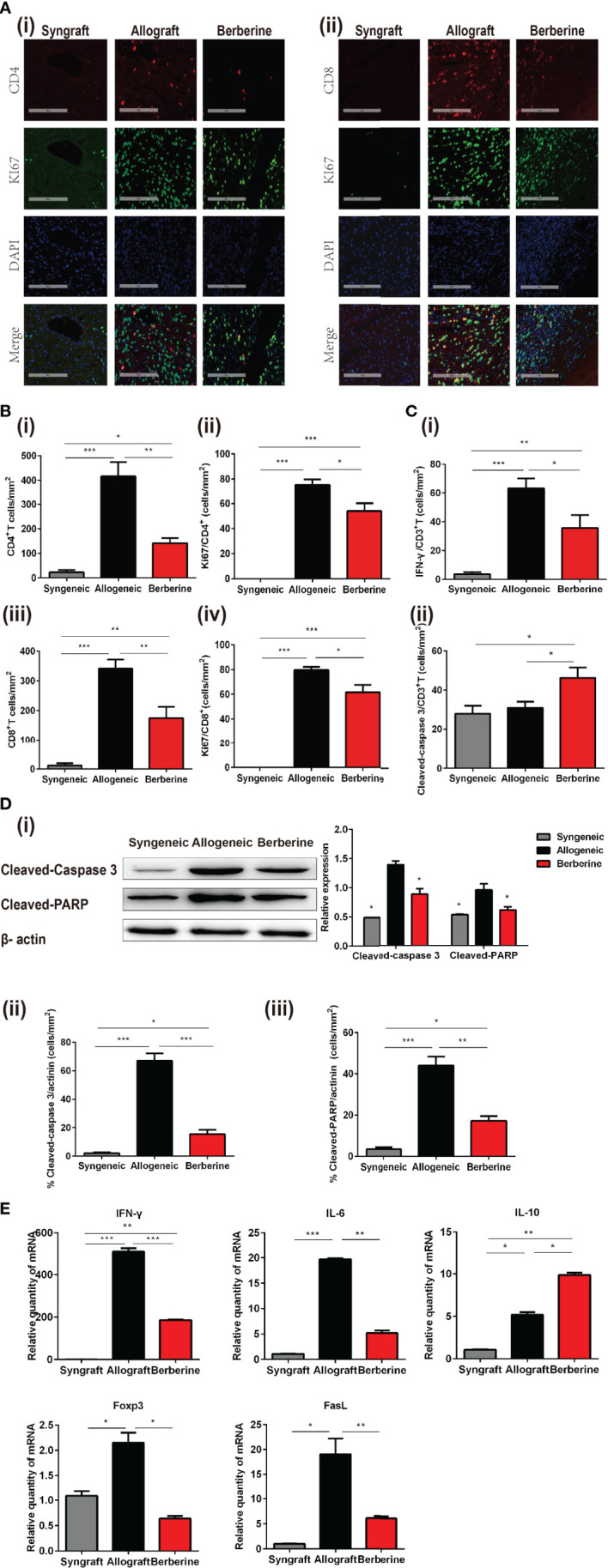
Phenotypic and functional characteristics of allograft-infiltrating CD4^+^ or CD8^+^ T cells. Allografts were recovered at POD 7, and POD 100 syngeneic grafts are shown for comparison. **(A)** (i) Immunofluorescent staining of CD4 (red), KI67 (green), and 4′,6-diamidino-2-phenylindole (DAPI, blue) in grafts. (ii) Immunofluorescent staining of CD8 (red), KI67 (green), and DAPI in grafts (Scale bar = 200 μm; original magnification: ×200). **(B)** Proportion and absolute number of graft-infiltrating (i) CD4^+^ T cells and their expression of (ii) KI67, and proportion and absolute number of graft-infiltrating (iii) CD8^+^ T cells and their expression of (iv) KI67 (n = 3 mice/group). **(C)** Proportion of (i) IFN-γ and (ii) cleaved-caspase-3 in graft-infiltrating CD3^+^ T cells (n = 3 mice/group). **(D)** (i) Cleaved-caspase-3 and cleaved-PARP protein expression in grafts. Myocardial cell apoptosis co-immunofluorescence staining and expression of (ii) cleaved-caspase-3 and (iii) cleaved-PARP (n = 3 mice/group). **(E)** Relative mRNA expression of *IFN-γ*, *IL-6, IL-10*, *Foxp3*, and *FasL* in grafts measured by qPCR (n = 3 mice/group). SPCs, spleen cells; LNCs, lymph node cells; POD, post-operative day. *p < 0.05, **p < 0.01, ***p < 0.001 compared to the normal saline-treated group.

The progression of T-helper 1 cell (Th1)-mediated acute cellular rejection involved mononuclear cell infiltration and proliferation. Next, we analyzed the infiltrating T cells in the graft at POD 7 *via* HE and immunofluorescence staining. As shown in **Figures 4A, B**, there was no obvious cellular infiltration in syngeneic grafts, whereas berberine treatment resulted in fewer CD4^+^ (**Figure 4Bi**) and CD8+ T cells (**Figure 4Biii**) in normal myocardial fibers compared to numbers in normal saline-treated mice. Co-immunofluorescent staining of CD4^+^ and the proliferation marker Ki67 or CD8^+^ and Ki67 showed that the proliferation of local effector T cells following normal saline treatment contributed to lesion-inducing effector T cell accumulation (**Figures 4A, Bii, iv**). In contrast, berberine treatment exhibited an inhibitory effect on lymphocyte proliferation, suggesting that the decreased effector T cell content could be attributed to the suppression of effector T cell infiltration. In addition, berberine treatment resulted in reduced expression of IFN-γ (**Figure 4Ci** and **Figure S1A**) in infiltrating CD3^+^ T cells, suggesting that the absence of graft rejection correlated with drastically decreased IFN-γ responses toward donor antigens. Moreover, following berberine treatment, allograft-infiltrating T cells expressed higher levels of the cleaved-caspase-3 apoptosis marker (**Figure 4Cii** and **Figure S1B**). In contrast, these markers were decreased in intra-graft CD3^+^ T cells of normal saline-treated mice, indicating that berberine treatment induced apoptosis in infiltrating T cells.

Graft rejection is associated with myocardial cell apoptosis. To gain further insight into the protective effect of berberine administration, the expression of cleaved-caspase-3 and cleaved-PARP in cardiomyocytes was measured by WB at POD 7. Compared to that with normal saline treatment, berberine treatment significantly inhibited the cleavage of caspase-3 and PARP (**Figure 4Di**). Actinin protein was mainly expressed by cardiomyocytes, whereas co-immunofluorescent staining of actinin and cleaved-caspase 3 (**Figure 4Dii** and **Figure S1C**) or actinin and cleaved-PARP (**Figure 4Diii** and **Figure S1D**) showed that berberine decreased cleaved-caspase-3 and cleaved-PARP in cardiomyocytes. No expression of cleaved-caspase-3 and cleaved-PARP was observed in syngeneic grafts. Lastly, to better understand the immune mechanisms involved in graft protection, we also measured a series of cytolytic and effector genes expressed in the grafts. Berberine treatment also led to reduced expression of *IFN-γ*, *IL-6*, *Foxp3*, and *FasL* mRNA compared to levels with normal saline treatment, suggesting downregulation of Th1 and cytotoxic T lymphocyte responses (**Figure 4E**).

### Berberine Induces T Cell Apoptosis *via* the Mitochondrial Apoptosis Pathway

**Figure 5:**
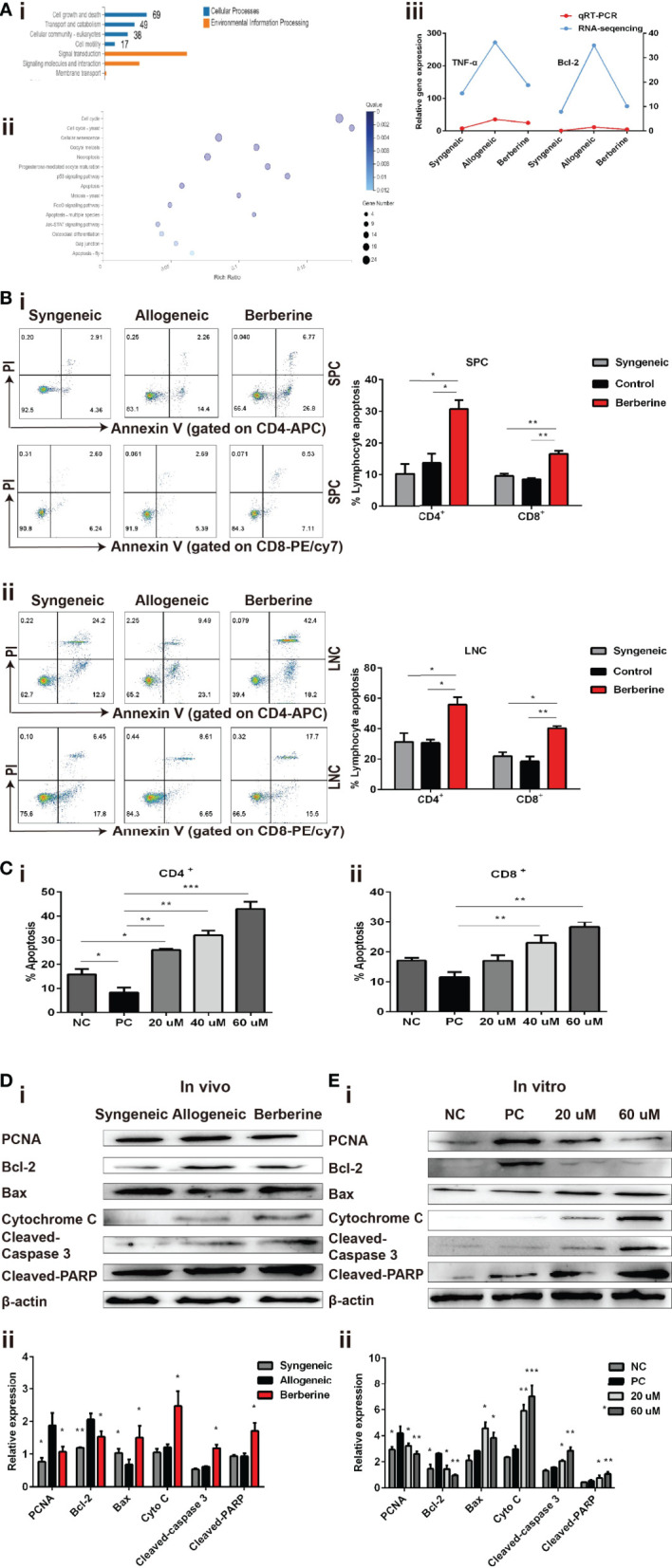
Berberine induces T cell apoptosis *via* the mitochondrial apoptosis pathway. **(A)** (i) KEGG functional categories of differentially expressed genes following berberine and saline treatment. The Y-axis represents the KEGG functional categories. (ii) KEGG analysis of the significantly altered signaling pathways in cell growth- and death-associated genes. The X-axis represents the rich ratio of the number of differentially expressed genes and the Y-axis represents the KEGG pathways. (iii) qPCR analysis of *Bcl-2* and *TNF-α* mRNA expression in SPCs collected from heart transplant recipients treated with berberine or not. **(B)** T cell apoptosis assays *in vivo*. (i) SPCs and (ii) LNCs were collected at POD 7. The percentages of apoptotic CD4^+^ and CD8^+^ T cells were determined by flow cytometry (n = 3 mice/group). **(C)** T cell apoptosis assay *in vitro*. T cells from naïve C57BL/6 mice were co-stimulated with anti-CD3/CD28 Abs in the absence or presence of berberine. The percentages of apoptotic (i) CD4^+^ and (ii) CD8^+^ T cells were determined by flow cytometry. **(D)** Berberine activates the mitochondrial apoptosis pathway *in vivo*. (i) Relative protein expression of Bcl-2, Bax, cytochrome c, cleaved-caspase-3, and cleaved-PARP in SPC. (ii) β-actin was used as a loading control (n = 3 mice/group), and OD values (relative to β-actin) are presented as means ± SEMs. *p < 0.05, **p < 0.01, ***p < 0.001 compared to the normal saline-treated group. **(E)** Berberine activates the mitochondrial apoptosis pathway *in vitro*. Relative protein expression of Bcl-2, Bax, cytochrome c, cleaved-caspase-3, and cleaved-PARP expression in CD3^+^ T cells. (ii) β-actin was used as a loading control; OD values (relative to β-actin) are presented as means ± SEMs. SPCs, spleen cells; LNCs, lymph node cells; POD, post-operative day. *p < 0.05, **p < 0.01, ***p < 0.001 compared to the PC group.

To further investigate the mechanism through which berberine decreases the number and inhibits the proliferation of effector T cells, SPCs were collected from the heart transplant recipient and used for RNA-Seq and bioinformatic analyses. The entire transcriptome screen identified a total of 1101 up- and downregulated mRNAs, including 69 genes involved in cell growth and death processes (**Figure 5Ai**). Kyoto Encyclopedia of Genes and Genomes analysis indicated that the enrichment of complex signaling pathways occurs following berberine treatment (**Figure 5Aii**). It has been reported that berberine induces apoptosis in tumor cells (24). In line with this result, our RNA-seq data showed an effect of berberine on various genes associated with the apoptotic pathway. For example, Bcl-2, an anti-apoptosis protein, was significantly decreased in berberine-treated mice compared to that in normal saline-treated mice (**Figure 5Aiii**). This suggests that berberine decreases the number and inhibits the proliferation of effector T cells by inducing cell apoptosis.

Subsequently, SPCs and LNCs were collected at POD 7 from the recipients in each treatment group and stained using an apoptosis kit. Compared to that with normal saline treatment, significantly higher apoptosis of CD4^+^ and CD8^+^ cells were observed in both SPCs (**Figure 5Bi**) and LNCs (**Figure 5Bii**) after berberine treatment. Similar results were obtained after stimulation with anti-CD3/CD28 Abs *in vitro*. Compared to that in the PBS group, the number of apoptotic CD4^+^ (**Figure 5Ci**) and CD8^+^ (**Figure 5Cii**) T cells increased significantly in the berberine group and berberine treatment showed a dose-dependent effect on apoptosis.

Apoptosis signaling is reportedly associated with mitochondrial damage. To better understand the effect of berberine on immunity and its involvement in T cell apoptosis, we examined whether it would have an impact on the mitochondrial apoptosis pathway in recipient SPCs. As shown in **Figure 5D**, berberine treatment effectively increased the expression of Bax, cytochrome c, cleaved-caspase-3, and cleaved-PARP, as compared to that in the control group, and the same pattern was observed in the syngeneic group. Moreover, our *in vivo* data showed that PCNA expression was significantly decreased following berberine treatment compared to that with normal saline treatment. Consistent with the *in vivo* data, the expression of these proteins in T cells was measured using WB analysis 2 days after T cells were stimulated *in vitro* with anti-CD3/CD28 Abs in the absence or presence of berberine (**Figure 5E**). Our results demonstrated a dose-dependent suppression of PCNA and Bcl-2 expression induced by berberine, which was accompanied by the upregulation of Bax, cytochrome c, cleaved-caspase-3, and cleaved-PARP expression. These findings indicate that berberine-induced T cell apoptosis could be attributed to activation of the mitochondrial apoptosis signaling pathway.

### Berberine Inhibits T Cell Activation, Proliferation, and Proinflammatory Cytokine Production *In Vitro*

**Figure 6:**
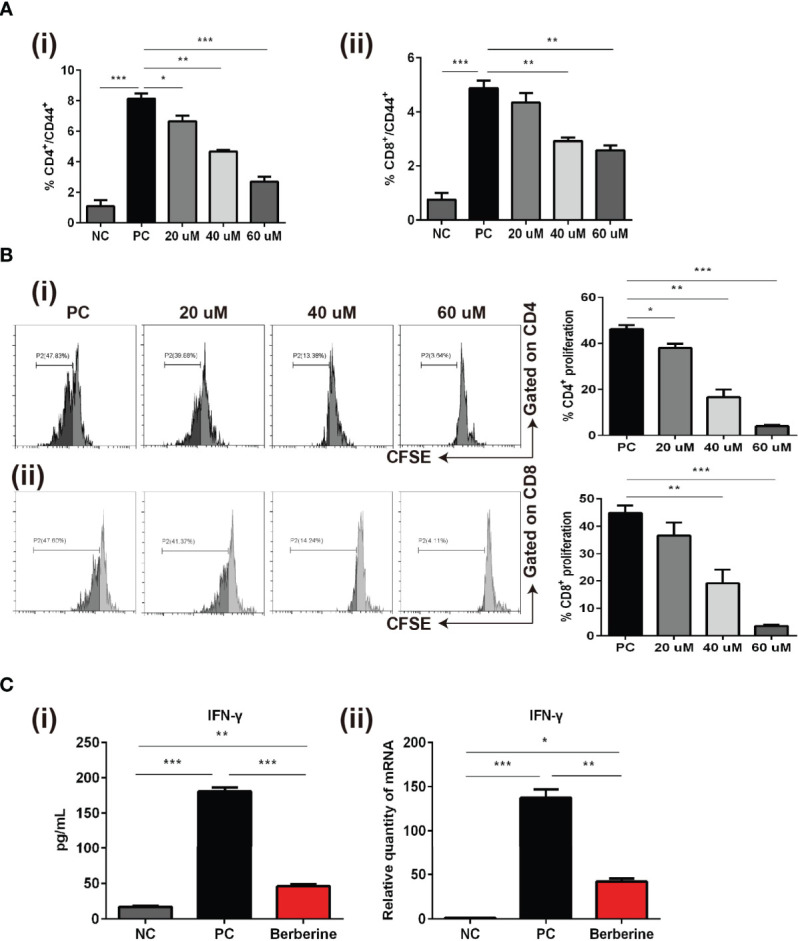
The effects of berberine on CD4^+^ and CD8^+^ T cell apoptosis, activation, and proliferation *in vitro*. **(A)** T cell activation assay. The percentages of (i) CD4^+^CD44^+^ and (ii) CD8^+^CD44^+^ T cells were determined by flow cytometry. **(B)** T cell proliferation assay. T cells from naïve C57BL/6 mice were labeled with CFSE and then co-stimulated with anti-CD3/CD28 Abs in the absence or presence of berberine. After 3 days of co-culture, (i) CD4^+^ and (ii) CD8^+^ T cell division was determined by flow cytometry. **(C)** (i) Supernatant levels of IFN-γ measured by ELISA, and (ii) mRNA expression of *IFN-γ* in T cells measured by qPCR. *p < 0.05, **p < 0.01, ***p < 0.001 compared to the (Positive Control) PC group.

Whereas we indicated that berberine suppressed T cell activation and reduced the number of effector T cells *in vivo*, we also investigated whether berberine would suppress T cell activation, proliferation, and effector cytokine secretion *in vitro*. Naïve SPCs were collected and processed to single-cell suspensions and stimulated with anti-CD3/CD28 Abs in the absence (PBS) or presence of berberine at low (20 mM), medium (40 mM) or high (60 mM) concentrations. After 24 h, the cells were harvested and CD4^+^ and CD8^+^ T cell activation was detected. CD44^+^ expression was significantly inhibited by berberine in both CD4^+^ and CD8^+^ T cells compared to that in the PBS group (**Figure 6A**). T cells from C57BL/6 mice were stained with CFSE and stimulated with anti-CD3/CD28 Abs in the absence or presence of berberine (20–60 mM) for 2 days. We found that berberine significantly suppressed CD4^+^ and CD8^+^ T cell proliferation in a dose-dependent manner (**Figure 6B**). Furthermore, we collected the supernatant and used ELISA to measure the levels of IFN-γ. As shown in **Figure 6C**, berberine significantly lowered IFN-γ levels, which is in line with the effects of berberine on T cells.

## Discussion

Immunosuppressive drugs are widely required for the prevention of both acute and chronic transplantation rejection; however, allograft survival is still limited owing to the toxicity and other side effects associated with global immunosuppression. Therefore, it is compelling to explore new therapeutic drugs capable of suppressing allograft rejection that exhibit fewer side effects. Berberine, a traditional Chinese medicine, exerts anti-inflammatory effects. Furthermore, multiple studies have shown that berberine has multiple effects on immune cell populations, such as i) inducing DC apoptosis *in vitro* (12), ii) suppressing the macrophage proinflammatory response *via* the AMPK pathway (15), iii) regulating natural killer cell-mediated cytotoxicity in colitis (25), and iv) suppressing Th17 and Th1 T cell differentiation (11). Using a mouse model of heart transplantation, we showed for the first time that berberine prolongs mouse allograft survival with no significant negative effects.

Moreover, *in vivo* acute or sub-acute toxicity tests suggested that a dose of 10 mg/kg of berberine appears to produce no toxic-related side effects (26, 27). However, in the present study, berberine significantly increased perioperative mortality in transplanted mice treated with high doses of berberine (>5 mg/kg). The decreased tolerance to berberine might be partially explained by the vast physiological changes following a major surgery such as cardiac transplantation. Clinical reports have shown that long-term treatment with berberine is safe and does not lead to any clinical deterioration and/or changes in creatinine levels and liver function (28). Congruent with these results, no obvious kidney or liver toxicity was observed following our administration regimen.

In the initial immune responses, T cells make a critical difference (29–31) and the secondary lymphoid organs are employed to activate T cells, influencing the process of graft rejection when they migrate to the transplanted organ. Specifically, tissue destruction and ultimate allograft rejection are associated with a high level of proinflammatory cytokines, which are produced by the infiltrating effector T cells. Therefore, a decrease in alloreactive T cells would benefit allograft protection. Our study demonstrated that berberine significantly decreases effector T cell expansion in peripheral immune organs.

The rejection of transplanted BALB/c mouse hearts in C57BL/6 mice is considered to occur *via* a T lymphocyte-mediated immune response. In general, increased graft-infiltrating T cells and type-1 cytokine (IFN-γ) production by splenic CD4^+^ and CD8^+^ T cells in the allografts are associated with acute rejection of the transplanted organ in rodents (32) and humans (33). In the present study, we demonstrated that berberine significantly suppressed CD4^+^ and CD8^+^ T cell proliferation, which was correlated with Th1-mediated acute rejection. Additionally, CD4^+^ T cells secrete cytokines, such as IFN-γ, to efficiently prime Th1 and cytotoxic CD8^+^ T cell responses (34). The absence of rejection was correlated with a drastic reduction in CD8^+^ T cell alloreactivity, which manifested through their inability to produce IFN-γ and display potent cytotoxic functions, as revealed by the downregulated expression of FasL when challenged by donor antigens (35). The present study demonstrated that berberine blocks IFN-γ production in CD3^+^ T cells, which was correlated with the defects in Th1 cells and helped in the prevention of immune rejection occurrence.

A decrease in T cell proportions in recipients is likely to result in a decrease in T-cell responses to alloantigens, thereby protecting the graft from rejection and inducing transplantation tolerance (36). Additionally, CD8^+^ T cells can directly induce myocardial cell apoptosis *via* the Fas/FasL pathway (37). The Fas-FasL system is involved in the cytotoxic T lymphocyte-induced killing of target cells (38, 39), which might lead to programmed cell death, as shown by their increased expression of apoptotic markers (active caspase-3) and their inability to survive in a lymphopenic environment (35). In the present study, we showed that berberine treatment decreased the generation of FasL in the allograft, which was a key component in the protective effect on allogeneic grafts. The effect on myocardial cells was associated to the low expression of cleaved-caspase-3 and cleaved-PARP. This was consistent with the effects of the clinical immunosuppressant cyclosporin A (CsA), which is widely used in organ transplantation to suppress allograft rejection. It has been reported that CsA treatment protects the allograft by inhibiting the expression of Bcl-2 in ventricular tissue (40). In a C57BL/10-to-C3H mouse heart transplantation model, recipients treated with 20 mg/kg of CsA from POD 0 to POD 7 showed significantly prolonged heart allograft survival to 15 days (41). Further, berberine inhibited the expression of genes encoding proinflammatory cytokines, including IFN-γ, TNF-α, and IL-6, but not Foxp 3, in heart allografts. Thus, we showed that berberine inhibits allograft rejection by suppressing myocardial cell apoptosis and the expression of proinflammatory cytokines.

Published work has shown that CD4^+^ T cells secrete cytokines, such as IL-2, to stimulate the proliferation and differentiation of cytotoxic T cells. CD44 and CD69 are an adhesion molecule and a type II membrane protein, respectively, and both of them are activation markers, which can be rapidly expressed on T cells after stimulation of the T cell receptor (42, 43). In heart transplant patients, more than 15% of T cells in peripheral blood are classified as CD8^+^CD69^+^ and mediate strong rejection (44). Histological observation indicated that the infiltration of T cells in the myocardium of patients with heart transplant rejection show CD69^+^ activation and perforin activity (45). In the present study, we showed that berberine could inhibit the proliferation of alloreactive T cells in response to stimulation with allo-antigens, as well as anti-CD3/CD28 Abs. Transplantation studies have indicated that CD8^+^ T cells can evade the immunosuppressive effects of immunosuppressors, such as cyclosporine and rapamycin, suggesting that CD8^+^ T cells might be involved in the development of chronic rejection. Therefore, it is necessary to determine the number of effector CD4^+^ and CD8^+^ T cells in patients and design a reasonable immunotherapy plan (46).

However, it remains controversial whether berberine decreases effector T cells by inducing apoptosis or through a different method. In accordance with the RNA-Seq results, pharmaceutical elimination of CD4^+^ and CD8^+^ T cells was therapeutically beneficial for allograft survival and simultaneously resulted in a significant increase in T cell apoptosis within the LNC and SPC populations of recipient mice. Furthermore, *in vitro* berberine treatment was shown to exhibit a dose-dependent effect on T cell apoptosis, which is in agreement with some reports that revealed the capacity of berberine to induce tumor apoptosis (8, 47, 48) *via* reactive oxygen species (ROS)-generating mechanisms, which involve the Fas/FasL-dependent pathway. Other studies have reported that berberine inhibits the proliferation of human peripheral T lymphocytes in response to mitogens or polyclonal activators through non-apoptotic mechanisms, such as cell cycle arrest (49). Further research is needed to explore the molecular mechanisms underlying berberine-induced apoptosis and cell cycle arrest in T cells.

A previous study demonstrated that Bcl-2 can suppress apoptosis in various cell lines (50). Furthermore, it has been reported that the overexpression of apoptosis inhibitors in recipient mice could reverse transplantation tolerance induced by a monoclonal antibody (36). In a preliminary study, the downregulation of Bcl-2 and upregulation of Bax were triggered following berberine treatment and caspase-3 and PARP were activated in T cells, which implies that the mitochondrial apoptosis pathway might be involved in berberine-associated T cell apoptosis. An *in vitro* study showed that the addition of caspase inhibitors, such as caspase-3 and caspase-8 inhibitors, can reverse the apoptosis induced by berberine in hepatoma (8). This implies that berberine induces apoptosis *via* the mitochondria-dependent pathway, which is consistent with the results of the present study.

Nevertheless, this study has a few limitations. First, the mechanism through which berberine regulates caspase-mediated apoptosis was not thoroughly investigated. However, previous studies explored this aspect in various cell lines. It has been suggested that berberine induces colorectal tumor cell apoptosis by activating the JNK/p38 MAPK pathway, as well as the production of ROS (51). This is supported by the capacity of berberine to induce ROS-mediated apoptosis in dendritic cells (12). The relative role of the ROS–caspase axis in T cells requires further investigation. The second limitation is represented by the lack of experiments investigating the regulatory role of TNF-α in T cell apoptosis or proliferation. According to the RNA-seq data, the apoptotic pathway associated with TNF-α expression was significantly decreased following berberine treatment. It has been reported that TNF-α activates the TNF receptor 2 signaling pathway, thus upregulating the expression of Bcl-2 in the T cell activation phase (52). This observation was supported by another study using TNF receptor 2-deficient mouse models wherein T cell accumulation was facilitated following primary stimulation (53). Therefore, the mechanism through which berberine regulates the TNF-α signaling pathway and T cell apoptosis requires further research.

The combination of innate immunity and adaptive immunity contributes to allograft rejection. However, the changes in monocytes and neutrophils in organ transplantation after berberine treatment have not been reported and need further study. The effect of berberine on neutrophils and monocytes has been reported in other disease models. We know that neutrophils and monocytes constitute the first line of defense for innate immunity. Monocytes in donor grafts play an important role in recruiting granulocytes and promoting allograft dysfunction (54). The infiltration of activated neutrophils in organs promotes the expression of pro-inflammatory factors and chemokines, such as CCR2 (C-C motif chemokine receptor 2), leading to organ dysfunction. It has been reported that berberine treatment can inhibit the expression of CCR2 and reduce the infiltration of neutrophils in the lungs, hearts, and kidneys of septic mice (55). The protective effect of berberine on the vascular endothelium has been recognized. The dysfunction caused by the adhesion of monocytes to the vascular endothelium is a prerequisite for the induction of atherosclerosis, hypertension, and other diseases (56, 57). Berberine treatment can reduce the expression of vascular endothelial adhesion molecules and suppress the adhesion of monocytes. Cyclooxygenase 2 (COX-2) plays a crucial role in promoting atherosclerosis. Berberine treatment can significantly inhibit the expression of COX-2 in human peripheral blood mononuclear cells through the ERK1/2 signaling pathway. In addition, berberine can also inhibit the expression of JNK in peripheral blood, which might also be one of the mechanisms to inhibit the progression of inflammation and atherosclerosis.

In summary, we demonstrated that berberine prolongs organ allograft survival, inhibits CD4^+^ and CD8^+^ T cell infiltration in the graft, and inhibits T cell proinflammatory cytokine production (IFN-γ) and their cytotoxic activity following transplantation. Berberine also suppressed T cell proliferation and activation both *in vivo* and *in vitro* while activating the mitochondria apoptosis pathway in T cells. The findings of this study together with the discussed studies provide baseline information for clinical trials using berberine as an effective immunosuppressive compound against allograft rejection.

## Data Availability Statement

The raw sequencing data reported in this manuscript have been deposited in the NCBI Sequence Read Archive (SRA) and are accessible through SRA accession number: PRJNA689623, or https://www.ncbi.nlm.nih.gov/sra/PRJNA689623.

## Ethics Statement

This study was carried out in accordance with the guidelines of the Animal Care and Use Committee and Ethics Committee of Xiamen University (Committee’s reference number: XMULAC20170243).

## Author Contributions

ZQ worked on experimental design. YM and GY performed animal experience. JG and CW performed WB and q-PCR. YM and HZ performed flow cytometry. YY and YC performed histology and immunofluorescence staining. YM performed data analysis and wrote the manuscript. HD and GZ contributed to data interpretation and reviewed the manuscript. All authors contributed to the article and approved the submitted version.

## Funding

The experimental studies were supported by the National Natural Science Foundation of China (81771271, 81800664, and 82070776), National Key R&D Program of China (2018YFA0108304), Natural Science Foundation of Hunan Province of China (2019JJ50842), Huxiang Young Talents of Hunan Province (2019RS2013), and College Students’ Innovative Entrepreneurial Training Plan Program (2020X0736).

## Conflict of Interest

The authors declare that the research was conducted in the absence of any commercial or financial relationships that could be construed as a potential conflict of interest.
