# Supplementary material for: Berberine Prolongs Mouse Heart Allograft Survival by Activating T Cell Apoptosis via the Mitochondrial Pathway
Source: Front Immunol. 2021 Feb 25;12:616074. doi: 10.3389/fimmu.2021.616074 (PMC7959711; doi:10.3389/fimmu.2021.616074)
Supplement: Supplementary file 1 [file DataSheet_1.pdf]

Supplementary Figure 1. Immunofluorescent staining of allografts harvested from each group at POD 7.

(A) Immunofluorescent staining of CD3 (green), IFN-γ (red), and 4′,6-diamidino-2-phenylindole (DAPI, blue, nuclei) in grafts (scale bar = 100 μm; original magnification: ×400). (B) Immunofluorescent staining of CD3 (green), cleaved-caspase-3 (red), and DAPI in grafts (scale bar = 100 μm; original magnification: ×400). (C) Immunofluorescent staining of cleaved-caspase-3 (red), actinin (green), and DAPI in grafts (scale bar = 100 μm; original magnification: 200×). (D) Immunofluorescent staining of cleaved-PARP (red), actinin (green), and DAPI in grafts (scale bar = 100 μm; original magnification: ×200).

POD = post-operative day.

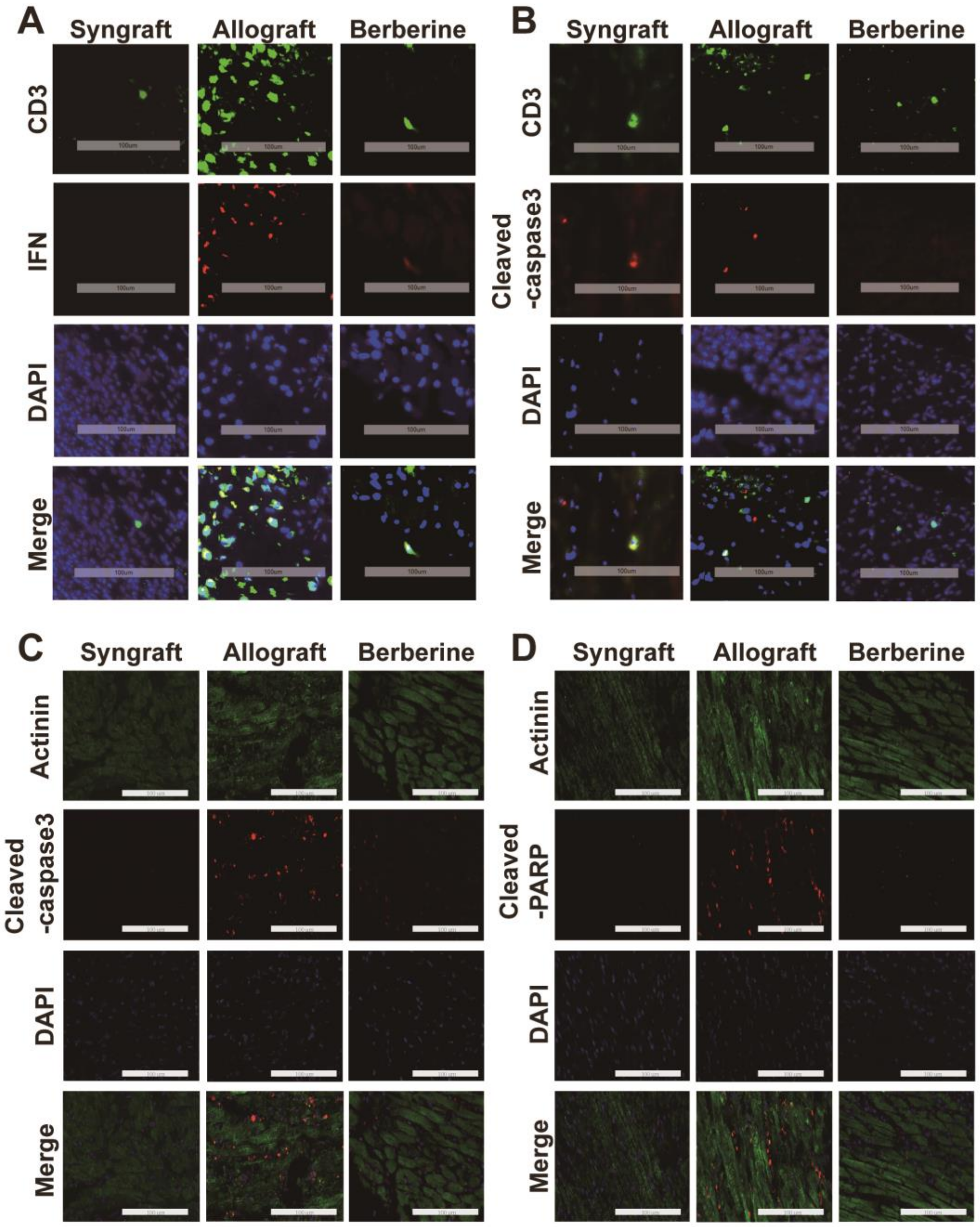

A
Syngraft
Allograft
Berberine
CD3
IFN
DAPI
Merge
B
Syngraft
Allograft
Berberine
CD3
Cleaved -caspase3
DAPI
Merge
C
Syngraft
Allograft
Berberine
Actinin
Cleaved -caspase3
DAPI
Merge
D
Syngraft
Allograft
Berberine
Actinin
Cleaved -PARP
DAPI
Merge
